# Imaging low-dimensional nanostructures by very low voltage scanning electron microscopy: ultra-shallow topography and depth-tunable material contrast

**DOI:** 10.1038/s41598-019-52690-9

**Published:** 2019-11-07

**Authors:** Laura Zarraoa, María U. González, Álvaro San Paulo

**Affiliations:** Instituto de Micro y Nanotecnología (IMN-CNM, CSIC), Isaac Newton 8, Tres Cantos, Spain

**Keywords:** Nanoscience and technology, Nanoscale materials, Techniques and instrumentation

## Abstract

We demonstrate the implications of very low voltage operation (<1 kV) of a scanning electron microscope for imaging low-dimensional nanostructures where standard voltages (2–5 kV) involve a beam penetration depth comparable to the cross-section of the nanostructures. In this common situation, image sharpness, contrast quality and resolution are severely limited by emission of secondary electrons far from the primary beam incidence point. Oppositely, very low voltage operation allows reducing the beam-specimen interaction to an extremely narrow and shallow region around the incidence point, enabling high-resolution and ultra-shallow topographic contrast imaging by high-angle backscattered electrons detection on the one hand, and depth-tunable material contrast imaging by low-angle backscattered electrons detection on the other. We describe the performance of these imaging approaches on silicon nanowires obtained by the vapor-liquid-solid mechanism. Our experimental results, supported by Monte Carlo simulations of backscattered electrons emission from the nanowires, reveal the self-assembly of gold-silica core-shell nanostructures at the nanowire tips without any ad-hoc thermal oxidation step. This result demonstrates the capacity of very low voltage operation to provide optimum sharpness, contrast and resolution in low-dimensional nanostructures and to gather information about nanoscaled core-shell conformations otherwise impossible to obtain by standard scanning electron microscopy alone.

## Introduction

Scanning electron microscopy (SEM) is currently one of the most extended characterization tools in all branches of nanoscience and nanotechnology. The continuous progress in instrumentation research and development delivers to the market increasingly powerful and user-friendly instruments. But SEM is probably such a commonly used technique that many users are not fully aware of the latest advances in imaging performance enabled by newly developed imaging methodologies. One of these probably overlooked developments is the ability of last-generation instruments to operate with very low incidence beam energies while keeping an extremely high resolution. The pioneering SEM researchers already realized around 80 years ago about the importance of reducing the incidence energy in order to fully exploit the unique imaging capacities of this technique^1^. However, high acceleration voltages (>5 kV) were used for decades because of the limitations of the available electromagnetic optics to provide high resolution at lower voltages. The technological advances produced by the end of last century motivated a great amount of research devoted to demonstrate the benefits of “low voltage” (LV) SEM, referring usually to the 1–5 kV range, mostly regarding the reduction of charge effects when imaging sensitive specimens^2–4^. This activity pushed the development of instruments that since several years ago allow routine operation in this LV range with sub-nanometer resolution for imaging a wide range of materials^5–8^. More recently, last generation instruments have extended extreme high resolution imaging to the “very low voltage” (VLV) range, denoting voltage values below the 1 kV limit^9–13^.

**Figure 1:**
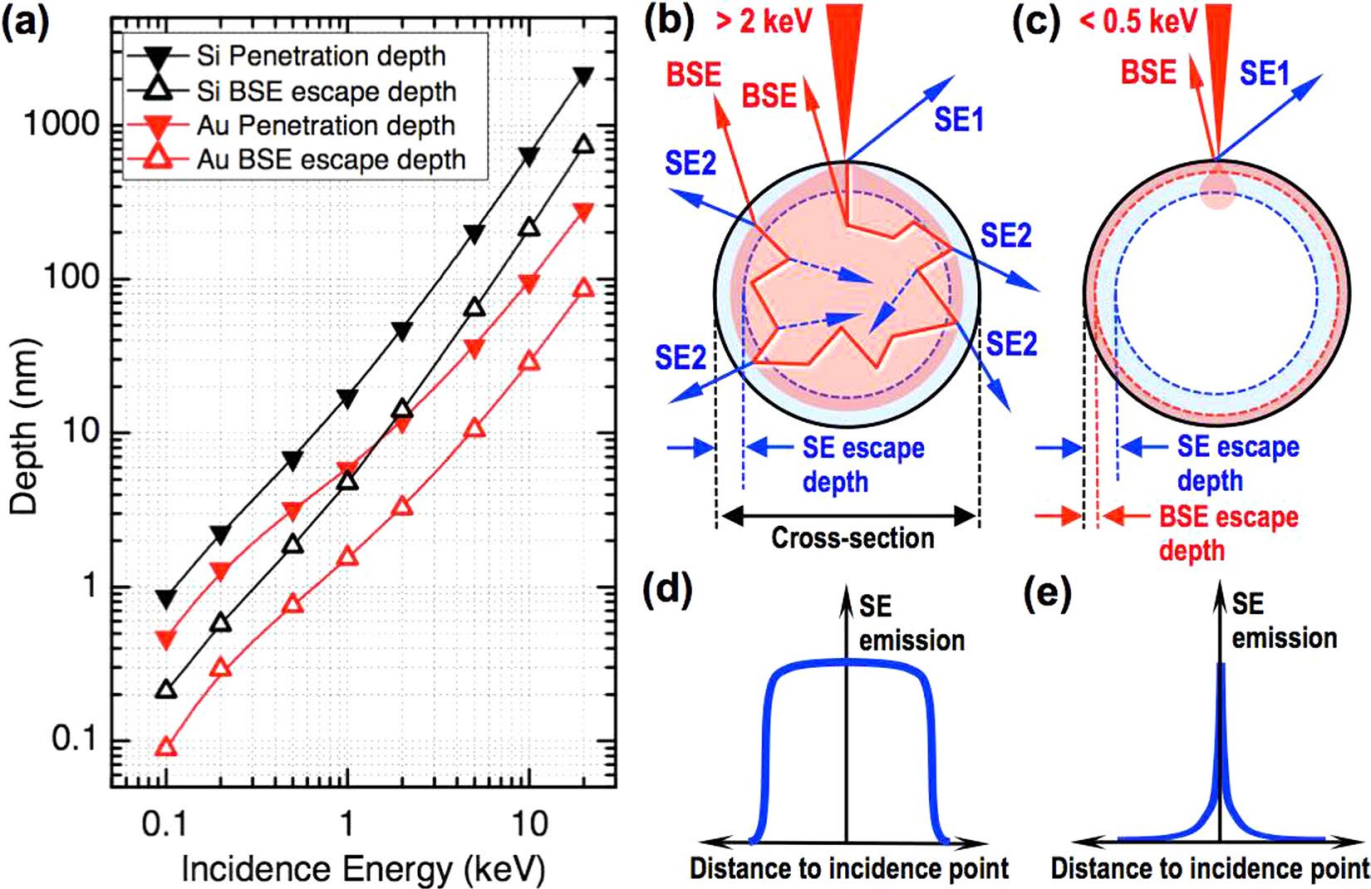
Effects of electron beam penetration depth on low-dimensional nanostructures as a function of incidence energy. (**a**) Monte Carlo simulations of penetration depth and BSE escape depth for Si and Au; (**b**,**c**) Schematic cross-sectional representations of beam incidence at high (**b**) and low (**c**) energies on a nanostructure indicating the interaction volume and the BSE/SE trajectories and escape depths. (**d**,**e**) Schematic plots of SE emission profiles at high (**d**) and low (**e**) energies, showing the qualitative effect of the SE2 background emission.

VLV-SEM imaging has obvious advantages for the characterization of insulating and fragile materials where charging effects are especially strong. But in addition, it has crucial implications for the characterization of low-dimensional nanostructures (thin layers, nanowires, nanoparticles) regardless of their electrical conductivity. The reason lies in the dependence of the beam penetration depth with the incidence energy. Above 2 keV, the beam penetration depth lays typically in the range from tens to hundreds of nm for a wide variety of materials, as calculated in Fig. 1a for the cases of Si (representing a relatively light element with Z = 14) and Au (representing a relatively heavy element with Z = 79). This implies that the beam can fill the whole cross-section of a nanostructure when its average diameter is comparable to the beam penetration depth, as schematically depicted in Fig. 1b. Remarkably, this range of sizes is precisely the typical range of a vast variety of low-dimensional nanostructures of interest in nanotechnology. Topographic contrast imaging is typically performed on these nanostructures in LV conditions by secondary electron (SE) detection, as in general SE emission yield is roughly independent of Z-number and SE escape depth is shallower than that of backscattered electrons (BSE) in such conditions^14^. The SE escape depth is indeed roughly independent of the beam incidence energy at low and very low voltages, and it varies from approximately 3 nm for light materials with average Z-number around 10 to approximately 10 nm for heavier materials with average Z-number around 40^15^. However, for low-dimensional nanostructures imaged in LV conditions, long-travel BSE can produce the emission of SE far from the incidence point, which are referred to as SE2^16^. As a consequence, electrons emitted from the nanostructures can come from any point on their surface far from the incidence point, with the subsequent loss of resolution and local information, as schematically depicted in Fig. 1b,d. On the contrary, VLV operation implies a much reduced beam-specimen interaction volume, so that both SE and BSE arise from a shallower region much more constrained to the incidence point, which optimizes resolution and contrast sharpness as shown in Fig. 1c,e.

Another key implication of VLV-SEM imaging of low-dimensional nanostructures is the ability to provide ultra-shallow topographic information virtually decoupled from material (Z-number) contrast. As the emission of SE2 is mediated by BSE, which is Z-number dependent, topographic contrast obtained by SE detection from low-dimensional nanostructures in conventional LV conditions can be strongly convolved to material contrast. This convolution can screen out topographic details that can be essential to understand the physical properties of the nanostructures under study. As an alternative, VLV operation enables the use of high-angle BSE detection for topographic contrast (see supplementary Fig. S1 for angle definitions). VLV conditions imply that BSE emerge from very superficial regions, carrying ultra-shallow topographic information. As the BSE scape depth does depend on the beam incidence energy, the BSE emission can become shallower and sharper than that of SE if the incidence energy is reduced sufficiently^2^. For instance, while the SE escape depth for Si is above 4 nm^15^, the BSE escape depth drops below 2 nm at 0.5 keV (Fig. 1a). Moreover, by collecting high-angle BSE it is possible to minimize the compositional contrast because of the reduced Z-number dependent contribution to BSE emission at high angles. On the other hand, material contrast is possible with VLV-SEM by detection of low-angle BSE. Furthermore, by varying the beam incidence energy it is possible to probe the material composition of nanostructures at tailored depths, providing depth-tunable material contrast. Low-angle BSE detection in the LV range produces material contrast from well deep beneath the surface because BSE escape depth can reach up to several tens of nm (Fig. 1a). In contrast, by varying the incidence energy in the VLV range it is possible to obtain depth-tunable material contrast imaging in very thin low-dimensional nanostructures where relevant conformational features occur at shallow depths of only a few nm.

In this work we show the implications of VLV-SEM imaging of low-dimensional nanostructures by using Si nanowires (NWs) grown by the vapor-liquid-solid (VLS) mechanism as a model system. This is indeed a particularly pertinent field of application of VLV-SEM given the trend in increasing complexity of Si NW based nanostructures reported in the last few years. Research on VLS grown Si NWs began to experience a rapidly growing interest around twenty years ago, being those early activities focused on understanding the growth mechanisms and on improving control of the NW properties^17,18^. The field has progressively evolved so that most of the activity reported during the last decade has turned towards specialized applications in multiples areas, from energy^19–23^ and healthcare^24–27^ to information and communications technologies^28–30^. In consequence, the interest on NW based nanostructures with increasing compositional and structural complexity is also growing. The VLS growth is indeed particularly well suited for exploring singular material combinations and/or complex structural features that are otherwise impossible to obtain. Some examples include various types of core-shell nanostructures^21,27,31^, functionalization with organic frameworks or 2D layered materials^22,32,33^ or conformal coatings of various inorganic materials^23,34^. The VLS growth mechanism is based on the use of metal catalyst nanoparticles (NP), typically Au, that assist the decomposition of the precursor gas and determine the growth site and diameter of the Si NWs, allowing for the hierarchical integration of NWs into complex devices^35^. We present imaging results by VLV-SEM that reveal that, under our particular processing conditions, it is possible to obtain the spontaneous formation of gold-silica core-shell nanostructures at the tip of the Si NWs instead of the pristine Au NP expected from the VLS mechanism alone. Image interpretation is supported by Monte Carlo simulations of beam-specimen interactions at the tip and body of the NWs. The self-assembly of core-shell nanostructures at the tips of NWs had never been detected before by SEM alone, pointing out the exceptional scientific potential of VLV-SEM for the routine characterization of low-dimensional nanostructures, in particular those with core-shell conformations.

## Methods

### Silicon nanowire growth

Si NWs were synthesized in a Nanoinnova “CVDCube” atmospheric pressure chemical vapor deposition (AP-CVD) system with SiCl_4_ as gas precursor. Colloidal Au nanoparticles (NP) with a nominal diameter of 150 nm (Sigma-Aldrich) were deposited on a Si (111) substrate to be used as catalyst. Before catalyst deposition, substrates were thoroughly cleaned, first in ultrasounds with sequential immersions in acetone, isopropyl alcohol and D.I. water, and then HF (5%) treated to remove native oxide (10 s immersion). Substrates were then coated by immersion in poly-L-lysine to improve Au NP adhesion, and afterwards they were immersed in the colloidal Au NP suspension. Si NWs were grown at a temperature of 825 °C in the tubular quartz reactor of the AP-CVD system. Liquid SiCl_4_ was used as a precursor by flowing 55 sccm of inert Ar gas through a bubbler kept at a constant temperature of 0 °C. H_2_ was introduced (10% in Argon) with a flow of 135 sccm. Growth time was 15 min. After that time, the precursor gases are purged with Ar from the tube for another 5 min, and then the tube is opened without reducing the temperature for a quick extraction of the samples and loading the next batch of substrates. The resulting nanowires have typical lengths around 10 μm, base diameters around 300 nm and tip diameters around 150 nm.

### Scanning electron microscopy and EDX spectroscopy

The microscope used in this work is a FEI Verios 460. In its conventional operation mode, as used for LV operation (2–5 kV), the instrument uses a detector named TLD (through the lens detector) for SE imaging with topographic contrast, and another one named MD (mirror detector) for BSE imaging with material contrast (supplementary Fig. S1). The TLD is placed in the last section of electromagnetic lenses at the bottom of the column, mounted inside the objective lens. To improve the SE collecting efficiency in the TLD, the end of the pole piece includes a group of electrostatic plates, forming a suction tube electrode; these plates are connected to a positive 70 V voltage which helps to attract SE towards the interior of the pole piece. In the superior end of the pole piece there are additional plates connected to a negative 15 V voltage, named the mirror electrode, which redirects the SE towards the TLD situated at an inner side of the polar piece. Higher inside the column, just on top of the objective lens, lies the MD which given such location captures low-angle BSE whose straight trajectory and high energy leave then unaffected by the mirror electrode.

VLV operation is performed by using beam deceleration (BD). This technique consists on applying a negative bias voltage to the sample holder so that the incident beam is decelerated when it reaches the sample surface. BD results in three advantageous effects (supplementary Fig. S1). First, the electric field resulting from the negative sample bias acts as an additional electrostatic (cathode) lens, reducing the beam diameter and thus improving spatial resolution^9^. Second, the electrons are decelerated just when reaching the sample surface, resulting in an effective incidence (or landing) energy that equals the acceleration voltage minus the beam deceleration voltage, and allowing the use of a high enough acceleration voltage that allows optimum performance of the column lenses. And third, the effect of the sample bias electric field on the electrons ejected from the sample improves both SE and BSE collection efficiency and redistributes SE and BSE for each detector. Low energy SE and low-angle BSE are driven into the column, not being collected by neither the TLD nor the MD. Mid-angle BSE carrying topography contrast are focused into the objective lens by the sample bias voltage and they collide with the mirror electrode, generating so-called SE3 (see supplementary Fig. S1b). These SE3 are collected by the TLD and used to produce an image with topographic information. However, this image may show some convolution to material contrast, as mid-angle BSE emission still depends on Z-number. Finally BD allows for the use of a third detector, named Concentric Backscattered Electrons detector (CBS), capable to detect BSE with the highest angles. This retractable detector is placed in the sample chamber, just beneath the pole piece. The CBS is composed of concentric detector rings which capture the highest angle BSE which are confined towards the column by the effect of the BD electric field. This CBS detector provides the most pure topographic contrast, as high-angle BSE emission is practically independent of Z-number. Finally, it is worth mentioning that besides the imaging implications just discussed, sample biasing techniques have also been recently applied in the context of focused electron beam induced deposition (FEBID) in order to improve the control over the dimensions of the growing 3D nanostructures by acting on the primary beam and the generated SE^36^. The general imaging parameters used in this work are the following: LV range acceleration voltage, 2 kV; VLV range acceleration voltage, 2 kV, with BD voltage of 1.5 kV, resulting in an incidence energy of 0.5 keV; current, 0.1 nA; working distance, 2.5 mm; dwell time, 10 μs; scanning mode, integration of 8 lines; image size, 1536 × 1024 pixels.

The FEI Verios 460 used in this work includes an EDX spectroscopy system from EDAX, with a retractable “Octane” EDX detector and specific software named “TEAM”. EDX maps (256 × 192 pixels) were obtained with an acceleration voltage of 5 kV and a current of 0.4 nA with a dwell time of 10 ms.

### Monte Carlo simulations

Monte Carlo simulations were performed with the free software CASINO V2.51. This program is a single scattering Monte Carlo simulation of electron trajectories in a solid. Two sets of simulations were carried out. The first one included simulations on pure Si and pure Au substrates at different incident beam energies. These calculations were performed in order to estimate the changes in penetration depth of the incident electrons and the escape depth of BSE when reducing the incidence energy and to compare the results with the escape depth for SE reported in the literature. For these calculations, the average values of the resulting distributions of primary beam penetration depth and BSE escape depth were considered. Simulations for each energy were repeated 10 times to ensure statistic significance and the number of electrons used for each simulation was 5000. The second set of simulations were oriented to explain the contrast variations observed in material contrast images at varying landing energies. These simulations included two different systems, one being a Si surface with a 2 nm thick native silicon oxide (SiO_2_) layer on top representing the Si NW body. The second system consisted on Au covered by a silicon oxide layer with a thickness from 0 to 20 nm, representing the NW Au tip encapsulated in a silicon oxide shell. For the silicon oxide a density of 2634 g/cm^3^ was used. The beam diameter was set to 1 nm and the number of electrons simulated was raised up to 500.000 in order to ensure consistency with the experimental conditions (100 pA current, 10 μs dwell time). Each simulation was carried out 10 separate times to obtain statistic significance.

## Results and Discussion

**Figure 2:**
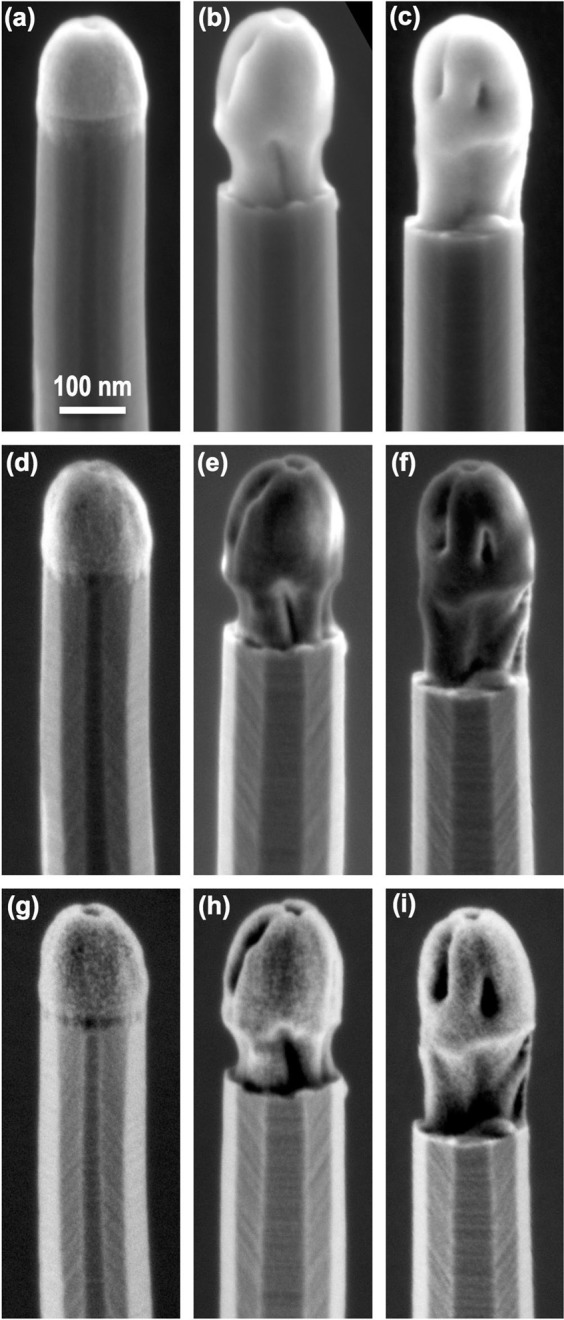
Topography contrast images obtained at different imaging conditions of Si NW tips with three different morphologies. (**a**–**c**) Images of Si NW with non-extruded, semi-extruded and fully-extruded tips obtained by SE detection with the TLD detector at 2 kV acceleration voltage. (**d**–**f**) Images of the tips of the same Si NWs obtained by mid-angle BSE detection with the TLD detector at 2 kV acceleration voltage with 1.5 kV deceleration voltage resulting in 0.5 keV incidence energy. (**g**–**i**) Images of the tips of the same Si NWs obtained by high-angle BSE detection with the CBS detector at the same voltage conditions as in (**d**–**f**).

Figure 2 displays a panel of images of the tips of three different Si NWs aiming at showing topographic information for different acquisition conditions. Each row of the panel shows images of the three NWs acquired in the same conditions. The first row corresponds to images acquired in conventional LV range (2 keV) by SE detection in the TLD detector. The second row presents images obtained in the VLV range (0.5 keV) by mid-angle BSE detection in the TLD detector. The third row shows images obtained also for VLV conditions (0.5 keV) but acquired by high-angle BSE detection in the CBS detector. Each column of the panel shows images of the same NW acquired in these three different conditions. Three differentiated morphologies are observed for the three NWs presented in Fig. 2, which are representative of the types of morphologies observed in general for the hundreds of nanowires imaged. An additional set of images is shown in the supplementary Fig. S2. For the three cases, the tip conformation is not consistent with a pristine Au NP at the tip of the NWs as reported in general for the VLS growth mechanism^17^. Instead, the images suggest a process of tip extrusion from the NW, so that the NP can be found in three conformations that we roughly define as non-extruded (Fig. 2a,d,g), partially extruded (Fig. 2b,e,h) and fully extruded (Fig. 2c,f,i).

Although this tip extrusion effect is clearly observed for all imaging conditions, important differences in contrast and sharpness are also appreciated between them. The images acquired at 2 kV (Fig. 2, first row) show a distinctive brighter contrast at the tips with respect to the NW body, which implies a larger emission of SE from the tip than from the NW body. This is consistent with a dominant presence of Au at the tip, as expected: although SE emission itself is roughly independent of Z-number^14^, SE emission here is greatly influenced by SE2 emission given the small NW diameter and the relatively large beam penetration depth at 2 kV, which reaches above 10 nm according to Fig. 1a; SE2 is mediated by BSE emerging at the surface far from the incidence point, and the yield of BSE is larger for heavier elements, as Au, producing the brighter contrast at the tip. As a consequence, many topographic details of the tip are masked by this composition dependent background contribution from SE2 emission. This result changes drastically when using the same detector but changing to 0.5 keV (Fig. 2, second row). Remarkably, image sharpness improves noticeably in all cases, and many details of the facets at the NW sidewalls are now more evident. The image contrast also shows a notable change, particularly for the semi-extruded and fully extruded morphologies: the contrast at the tip is now darker as compared to the NW body. It should be noticed that according to the calculations presented in Fig. 1, the penetration depth in Au changes from around 20 nm at 2 keV to around 3 nm at 0.5 keV, i.e., almost one order of magnitude. In consequence, at 0.5 keV the beam is probing a much shallower region of the NW tips. According to the previous arguments, the contrast inversion observed suggests the presence of a material lighter that Si at these shallower regions of the tips. Finally, another major change is observed when using the CBS detector at 0.5 keV (Fig. 2, third row). All topographic details, both at the NW body and at the tip, are most clearly observed in these conditions. In addition, the tips do not show any systematic brighter or darker background contrast with respect to the NW bodies. This means that no material-dependent contribution to the image contrast is present in this case, as expected from high-angle BSE detection.

**Figure 3:**
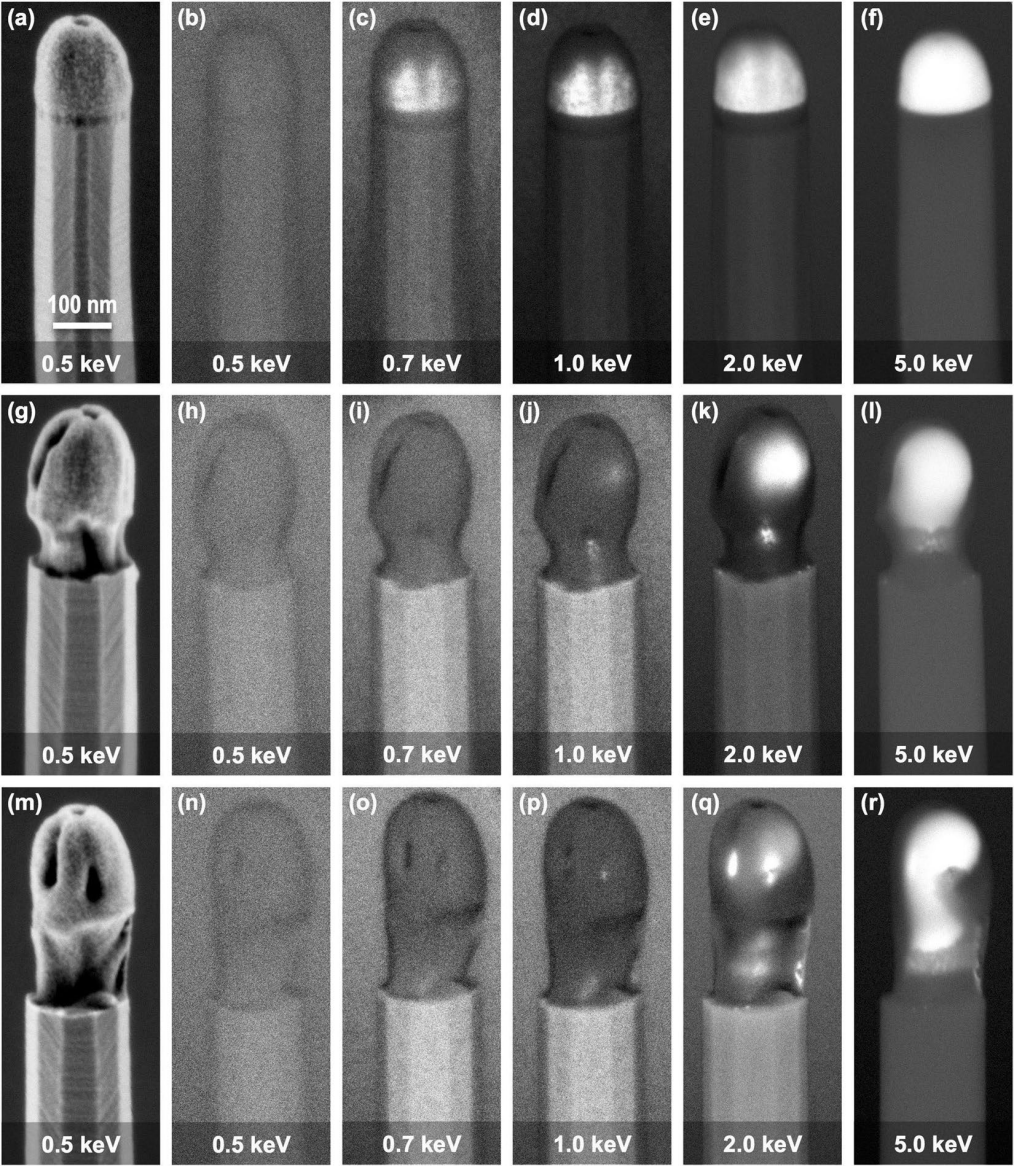
Material contrast images obtained at different incidence energies of Si NW tips with three different morphologies. (**a**,**g**,**m**) Ultra-shallow topography contrast images of the Si NWs with non-extruded (**a**), semi extruded (**b**) and fully extruded tip, obtained by high-angle BSE detection with the CBS detector at 2 kV acceleration voltage with 1.5 kV deceleration voltage resulting in 0.5 keV incidence energy (included again for reference). (**b**–**f**) Material contrast images of the non-extruded tip Si NW obtained by low-angle BSE detection with the MD detector at variable incidence energy without beam deceleration in all cases. (**h**–**l**) Material contrast images of the semi-extruded tip Si NW obtained in the same conditions as (**b**–**f**). (**n**–**r**) Material contrast images of the fully-extruded tip Si NW obtained in the same conditions as (**b**–**f**).

The results presented in Fig. 2, and in particular the observed contrast inversion effect, suggest a tip conformation more complex than a pristine Au NP at the tip of the Si NWs. In order to further investigate this conformation we acquired a series of images aiming at providing material contrast based on low-angle BSE detection with the MD detector. These series were obtained varying the landing energy from 0.5 to 5 keV, so that the resulting material contrast information comes from different depths for each image. No beam deceleration was used at any energy. According to calculations in Fig. 1, BSE emission depth at 0.5 keV is about 0.8 nm for Au and 2 nm for Si, while at 5 keV it increases to around 10 nm for Au and 60 nm for Si. This is a significant increase as compared to the NW tip radius, which has values in the range of 70–100 nm. Figure 3 shows the series of images acquired by low-angle BSE detection at variable landing energy for the same NWs presented in Fig. 2. Each row of the panel in Fig. 3 corresponds to each of the NW morphologies. As a reference, the first column shows the ultra-shallow topography contrast images of each NW obtained in VLV conditions (0.5 keV) by detection of high-angle BSE in the CBS detector. Each column from the second to the last one presents the material contrast images obtained by detection of low-angle BSE in the MD detector at landing energies of 0.5, 0.7, 1, 2 and 5 keV, respectively.

For the non-extruded tip morphology (Fig. 3, first row) a strong BSE emission from the tip is observed at the higher incidence energy (5 keV), producing a uniform and much brighter contrast at the tip with respect to the NW. As the energy is decreased, the tip-body contrast also decreases, becoming progressively less uniform and completely disappearing at 0.5 keV. For the semi-extruded tip morphology (Fig. 3, second row), the evolution of the tip-body contrast for decreasing energy shows some differences with respect to the non-extruded case. At the higher energies, a strong BSE emission from the tip and a large tip-body contrast are also observed, but the contrast within the tip is not as uniform as for the non-extruded case. But most remarkably, a contrast inversion is observed when energy is decreased, so that the tip becomes darker than the NW body when the energy changes from 2 to 1 keV. The contrast within the tip is actually quite irregular at these intermediate energies, being brighter than the body in some areas and darker in some others. At the lower landing energies, the contrast at the tip becomes uniformly darker than that at the NW body. Similar behavior is observed for the fully extruded morphology (Fig. 3, third row), with the exception that the contrast inversion occurs at a higher energy. At 5 keV there is strong BSE emission from the tip, but the irregular contrast reveals an intricate structure. When reducing the energy at 2 keV, the contrast inversion turns the tip predominantly darker than the NW, although brighter areas are still visible. At lower energy, the tip becomes uniformly darker than the NW as in the previous case, but this dark tip-NW contrast is stronger for the fully extruded morphology than for the other morphologies.

As the reduction in incidence energy implies a reduction of the BSE escape depth, the evolution of the tip-body contrast for decreasing energy observed in Fig. 3 can be interpreted in terms on changes in material composition from deeper to shallower regions of the tips. More precisely, the contrast inversion observed for the extruded morphologies suggests the encapsulation of the Au NP into a shell of a material lighter than Si. The spontaneous formation of an encapsulating layer at the tip of Si NWs has been rarely reported earlier, although some previous studies are compatible with the presence of a SiO_2_ shell surrounding the Au NP core^37,38^. In addition, the formation of encapsulating SiO_2_ structures around Si NWs has been previously observed by TEM as the result of intended thermal oxidation of the NWs by injecting an oxygen flow into the growth chamber after Si NW growth^39^. Our growth process does not include any controlled injection of oxygen during or after growth, but our protocol for extracting the samples from the tube furnace implies the exposition to ambient oxygen when the samples are still at high temperature, which can be expected to produce the observed morphologies (a detailed explanation is provided in the supplementary Fig. S3).

**Figure 4:**
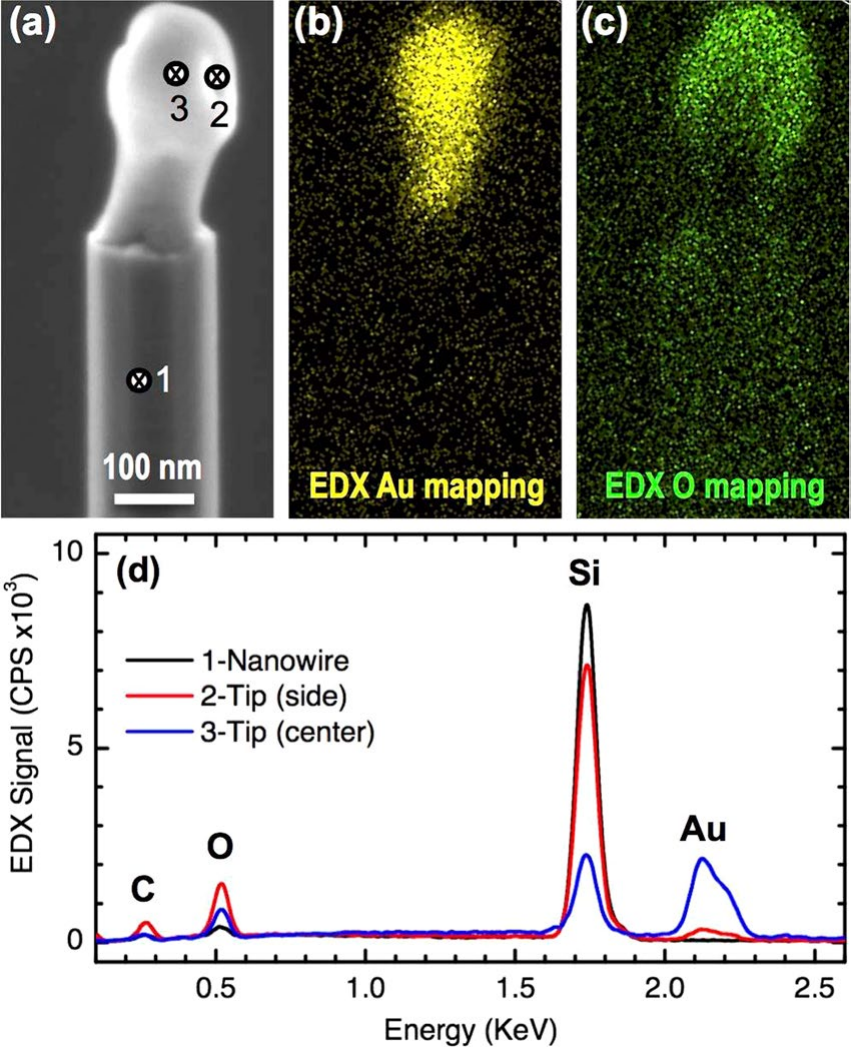
EDX element maps of a Si NW tip and spectra at different points of the tip. (**a**) Topography contrast image for reference (SE detection, TLD detector); (**b**) EDX gold map. (**c**) EDX oxygen map. (**d**) EDX spectra at the NW body (point 1), lateral side of the tip (2) and central part of the tip (3).

In order to provide further evidence of the encapsulation of the Au NPs into a silica shell for the case of the Si NWs studied here, we have performed EDX spectra and element mapping acquisition at the tips of the NWs. A representative example is shown in Fig. 4 for the case of the extruded tip morphology (equivalent results were obtained for all morphologies). The comparison between the topographic contrast image (Fig. 4a) with the Au map (Fig. 4b) confirms that Au concentrates in the central regions of the tip. However, the oxygen map (Fig. 4c) reveals that this element accumulates at the external regions of the tip, a result that is consistent with the presence of a SiO_2_ encapsulating shell. Si maps (not shown) are not meaningful because the Si substrate produces a strong background signal that masks the signal coming from the NWs. However, single spectra acquired at different points of the NW tips provide further indication of the presence of the silica shell surrounding the Au NP (Fig. 4d). The spectrum acquired at the center of the NW body (point 1) shows a strong Si Peak, a small presence of oxygen attributed to native oxide and no presence of Au. The spectrum acquired at the center of the tip (point 3) shows a much weaker presence of Si, a strong presence of Au and a significant presence of O. Finally, the spectrum acquired the tip side shows a significant presence of Si and O and a much reduced presence of Au. These results are fully consistent with the existence of a SiO_2_ shell around a Au NP core at the NW tips.

**Figure 5:**
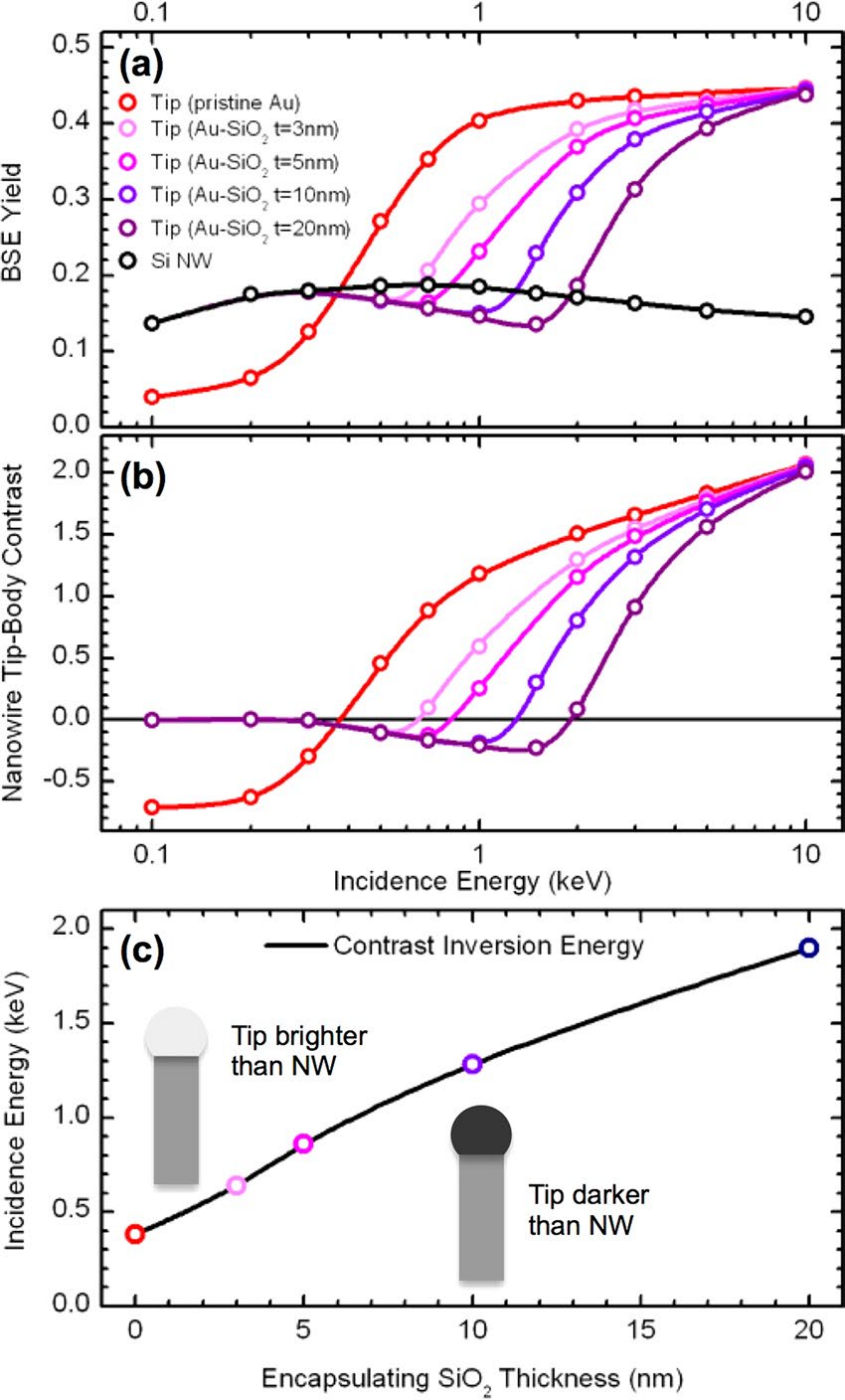
Monte Carlo simulations of BSE yield and NW tip-body material contrast from BSE detection. (**a**) Monte Carlo simulations of BSE yield versus incidence energy from the Si NW body (modeled as a Si substrate with 2 nm of native silicon oxide) and the NW tip (modeled as a Au substrate with a silicon oxide layer of 0, 3, 5, 10 and 20 nm of thickness). (**b**) Calculation of tip-body contrast versus incidence energy from the previous calculation of BSE yield. (**c**) Estimation of the incidence energy where the contrast inversion is produced as a function of the silicon oxide thickness at the tip.

We have performed Monte Carlo simulations with the objective of providing theoretical support to the interpretation of the depth-tuned material contrast images of Fig. 3 that points out to the presence of a silica shell encapsulating the Au NP at the NW tips. In the simulations, the Si NW body is modeled as a Si substrate covered by a 2 nm thick native silicon oxide layer, while the tips are modeled as a Au substrate covered by a silicon oxide layer with a varying thickness of 0, 3, 5, 10 and 20 nm, representing NW tips with different encapsulating silica shell thickness. In these simulations we calculate the variation of contrast between the tip and the NW body by firstly computing the changes in BSE emission yield (*η*) from each part for an incidence energy in the range from 10 to 0.1 keV. The results are presented in Fig. 5a. While the BSE yield varies slightly for the Si NW body, it is subject to important changes for the tip as energy decreases. At the higher energies, the BSE yield for the tip is around a factor of 3 higher than that of the Si body regardless of the silica shell thickness at the tip. As the energy decreases, the BSE yield at the tip also decreases. But remarkably, such decrease starts at higher energies for the tips with the thicker oxide shell. For all values of the silica shell thickness, there is a threshold energy for which the BSE yield at the tip becomes lower than that at the Si NW body: the thicker the silica shell, the larger the threshold energy. At the lower energies, all BSE yields converge to that of the Si NW body, except for the uncovered Au. These results can be interpreted by considering the dependence of the beam penetration depth and BSE emission depth with the landing energy: at high energies (>5 keV), the beam fully penetrates the silica shell at the tips, so that the Au core dominates the BSE emission regardless of the shell thickness. As the energy decreases, the beam probes more superficial regions, so that the BSE emission is progressively dominated by the silica layer, which implies a decreasing BSE yield because silica is much lighter than Au. A thicker silica shell implies that the beam interaction volume can be mostly confined to the shell at higher energies, which explains why the BSE yield drops at higher energies for tips with thicker shell. The fact that silica is lighter than Si (lower average Z-number) explains why the BSE yield reaches lower values at the tip than at the Si body for a given range of energies. In this range, the beam is mostly interacting with the lighter silica shell at the tips, while it is interacting with the heavier Si at the body of the NWs. At much reduced energies (<0.3 keV), the beam interaction volume is so shallow that it interacts mostly with the silicon oxide layer both at the tip (encapsulating shell) and at the NW body (native oxide), so that BSE yield converges to the same value.

The relative contrast between the tip and the NW body can be calculated from the BSE yield as *C* = (*η*_*TIP*_ − *η*_*BODY*_)/*η*_*TIP*_. The results are presented in Fig. 5b. At the higher energies, the tip-body contrast does not depend on the encapsulating silica shell, and it is the same as for the case of a non-encapsulated pure Au tip. Then, as landing energy decreases, the contrast is reduced more steeply and at higher energies for the tips with thicker silica shells. In all cases, an energy interval around 0.5–2 keV where the contrast is inverted (taking negative values) appears, but this range is wider and involves higher energies for the tips with thicker shells. Also for all cases, the contrast vanishes at very low energies (<0.3 keV). These calculations support the interpretation of the experimental images: contrast inversion happens as a consequence of the variation of the beam penetration depth for decreasing landing energy, which implies that at higher energies the beam interacts mostly with the core while at lower energies it interacts with the shell; the differences in BSE yield between the core and shell materials are thus the cause of the changes in contrast.

Besides supporting our interpretation of the imaging results, the simulations also allow estimating the thickness of the silica shell encapsulating the Au NPs. From the contrast calculation it is possible to determine the value of the incidence energy for which the contrast inversion happens, which we denote as “inversion energy”. We specifically define the inversion energy as the incidence energy for which the tip-body contrast changes from positive to negative as energy is decreased in Fig. 5b. Figure 5c shows a plot of the inversion energy as a function of the corresponding silica shell thickness. The resulting points, joined by an interpolating line, show a monotonic decrease of the inversion energy with the shell thickness. This line separates two regions in the graph, so that for a given thickness of the shell, a landing energy above the inversion energy line would result on a “tip-brighter-than-body” contrast, while a landing energy below the line would result on a “tip-darker-than-body” contrast. In order to estimate the silica shell thickness of the tips imaged in Fig. 3 we just need to consider what is the incidence energy at which the contrast inversion is observed. For the non-extruded tip morphology, no inversion is actually observed, and the tip-body contrast vanishes at 0.5 keV (Fig. 3, first row). This is the kind of behavior reproduced in the calculation of the tip-body contrast for the thinner value of the silica shell (3 nm) at the tip in Fig. 5b. Although a very small contrast inversion range is obtained theoretically, the range is likely masked by noise in the experimental images. According to these results, the silica shell thickness is below 3 nm for the non-extruded morphology. For the semi-extruded morphology, the contrast inversion is observed between the images acquired at 2 keV and 1 keV (Fig. 3, second row). According to Fig. 5c, this implies that the silica shell thickness at the tip varies between 7 and 20 nm. Finally, for the fully extruded morphology, the contrast inversion is observed between 5 keV and 2 keV (Fig. 3, third row), which indicates a shell thickness above 20 nm.

## Conclusions

In summary, in this work we have demonstrated crucial implications of VLV-SEM characterization of low dimensional nanostructures regarding the acquisition of images with ultra-shallow topographic contrast and depth-tunable material contrast. Ultra-shallow topographic contrast is obtained by detection of high-angle BSE while using beam deceleration, and it provides morphological information virtually decoupled from material contrast. This approach provides exceptional imaging power to gather information with extreme high resolution not only about the size of low-dimensional nanostructures, as conventional SEM already does, but also about much smaller and purely superficial features otherwise very difficult to observe such as ultra fine roughness, superficial defects, tiny amounts of adsorbed materials or highly dense small-diameter porosity, which often determine the physical, chemical or biological behaviour of the systems under study. Depth-tunable material contrast is achieved by detection of low-angle BSE without beam deceleration at varying incidence energy, so that energy values starting from the VLV range provide material contrast from the most superficial layers and allow probing conformational changes from only a few nm deep, while higher voltages offer compositional information of deeper features. This method can be remarkably useful for the characterization of core-shell nanostructures, as it provides a simple way to detect the presence of this conformation; to probe the uniformity of the shell layer; and even to estimate shell thickness. The application of such imaging approaches to VLS grown Si NWs as a model system has proved the self-assembly of Au-SiO_2_ core-shell nanostructures at the tips of the NWs that we attribute to the particular after-growth cooling conditions used, which favor the oxidation of the silicon segregated from the Au-Si NP alloy. The main evidences of such Au-SiO_2_ core-shell conformations at the Si NWs tips are the singular extruded morphologies observed in the ultra-shallow topographic contrast images obtained by high-angle BSE detection at 0.5 keV and the contrast inversion effect observed in the depth-tuned material contrast images obtained by low-angle BSE detection at variable landing energy in the 0.5 to 5 keV range. The images reveal an intricate structure of the tips, where the silica shell thickness is correlated to the degree of extrusion, being thicker for the more extruded tips. The shell thickness is much variable in all cases, being less uniform for the more extruded tips. This lack of uniformity produces that the contrast inversion does not happen abruptly at a given landing energy, but instead there is a range of energies where brighter and darker contrasts coexist at the NW tips. A perfectly uniform shell thickness would produce a much sharper transition, so that the tip-body contrast would change from fully brighter to fully darker at some specific energy depending on the thickness. These nanostructures can be relevant for Si NW applications where the conformation of the tip is significant, such as whenever the plasmonic properties of the Au NPs are exploited or whenever the Au NPs need to be removed by etching procedures that depend on tip composition. These results demonstrate the great potential of VLV-SEM for the characterization of low dimensional nanostructures in general and core-shell nanoparticles in particular, providing both superficial and structural information which is not accessible by characterization with conventional SEM alone.

## Supplementary information

Supplementary Information
