# Supplementary material for: Imaging low-dimensional nanostructures by very low voltage scanning electron microscopy: ultra-shallow topography and depth-tunable material contrast
Source: Sci Rep. 2019 Nov 7;9:16263. doi: 10.1038/s41598-019-52690-9 (PMC6838169; doi:10.1038/s41598-019-52690-9)
Supplement: Supplementary file 1 — Supplementary Information [file 41598_2019_52690_MOESM1_ESM.pdf]

Supplementary Information

Imaging low-dimensional nanostructures by very low voltage scanning electron microscopy: ultra-shallow topography and depth-tunable material contrast

Laura Zarraoa, María U. González, Álvaro San Paulo*

Instituto de Micro y Nanotecnología (IMN-CNM, CSIC)
Isaac Newton 8, Tres Cantos, Spain

*Corresponding authors email: alvaro.sanpaulo@csic.es

CONTENTS

Figure S1: Representation of the electron detection schemes used in this work.

Figure S2: Ultra-shallow topography images of a set of ten nanowires with tips showing different degrees of extrusion.

Figure S3: Representation of the formation process of the gold-silica core-shell nanostructures at the tip of Si NWs.

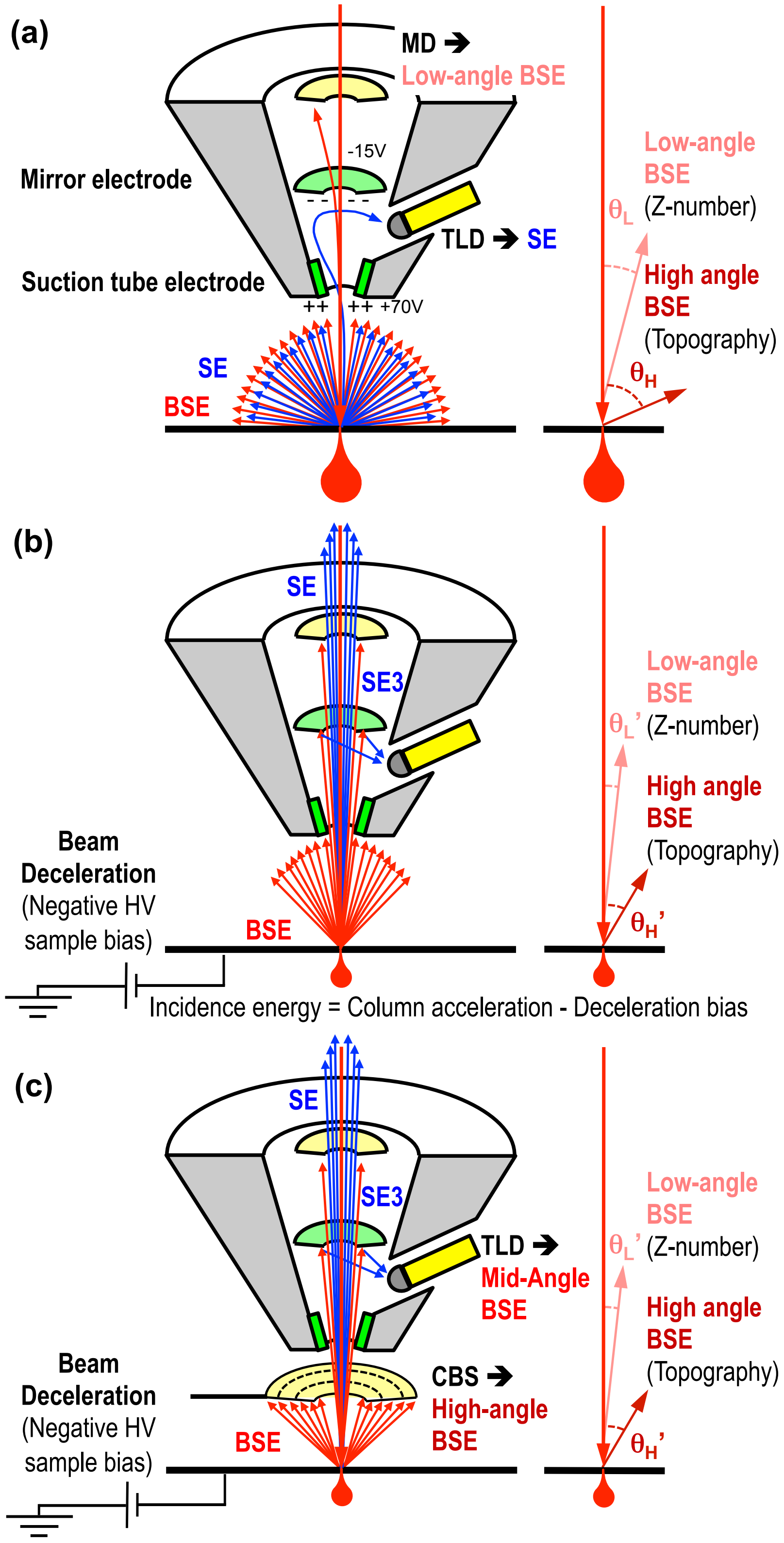

Supplementary Figure S1. Representation of the electron detection schemes used in this work. (**a**) Conventional scheme used for topography contrast imaging in LV conditions by SE detection in the TLD detector, and material (Z-number) contrast imaging in LV and VLV conditions by low-angle BSE detection in the MD detector; (**b**) Effects of beam deceleration bias: Incidence energy is reduced, and both SE and BSE are deflected towards the column; (**c**) Detection scheme used for ultra-shallow topography contrast imaging in VLV conditions by high-angle BSE detection in the CBS detector.

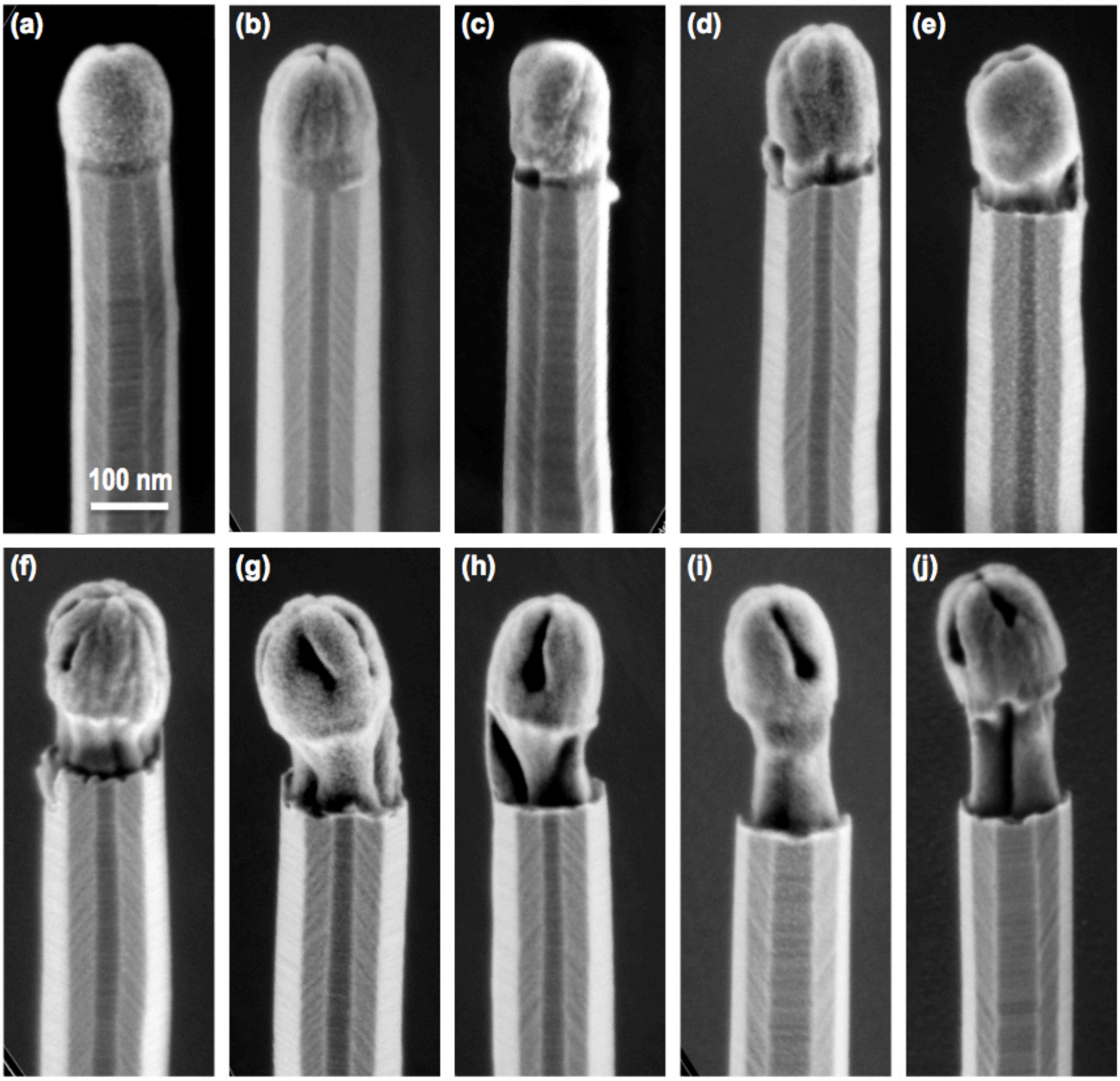

Supplementary Figure S2. Ultra-shallow topography images of a set of ten nanowires with tips showing different degrees of extrusion. All images were acquired in the same VLV conditions: high-angle BSE detection with the CBS detector at 2 kV acceleration voltage with 1.5 kV deceleration voltage resulting in 0.5 keV incidence energy.

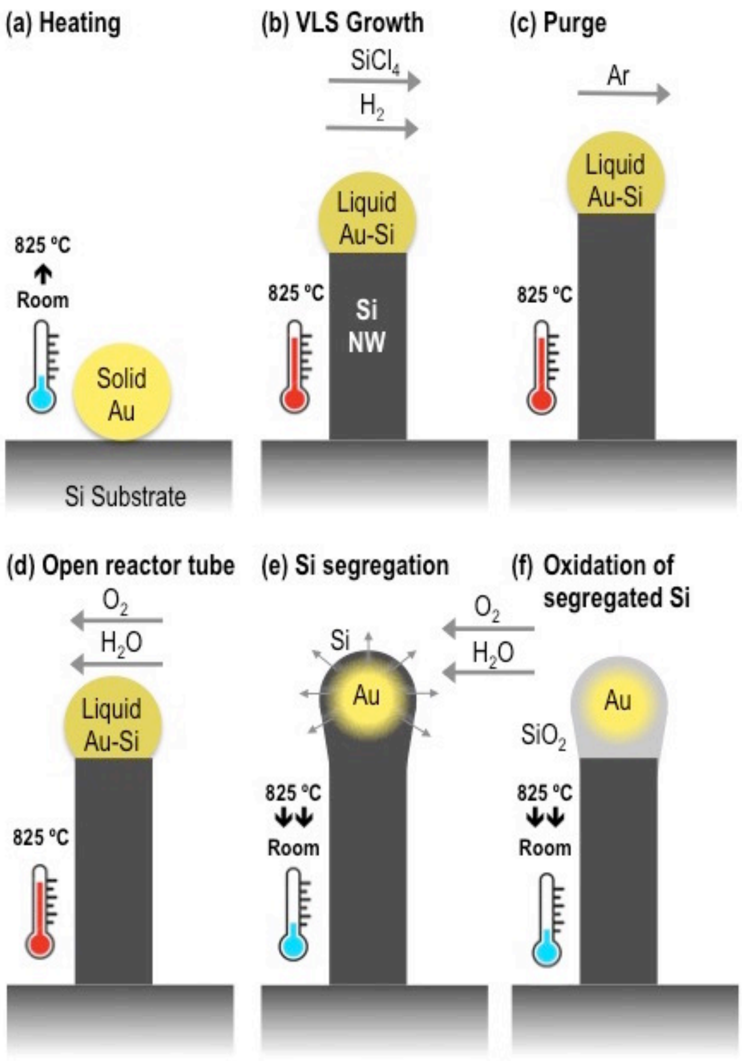

Supplementary Figure S3. Representation of the formation process of the gold-silica core-shell nanostructures at the tip of Si NWs. An explanation for the formation of the encapsulating silica shell at the NW tips can be found in our protocol for extracting the samples from the tube furnace once the growth reaction is finished. The completion of the synthesis process **(a, b)** is carried out first by stopping the flow of reactive gases, then purging the reactor tube for 5 minutes with inert Ar **(c)**, and finally extracting the samples from the reactor while maintaining the temperature of the furnace equal to the growth temperature **(c, d, e)**. At the time of opening the furnace **(d)**, the NPs formed by the Au-Si alloy remain in the liquid state, and according to the binary phase diagram, they maintain a proportion of remaining Si that has not been incorporated into the NW. Given the abrupt temperature change experienced by these yet liquid NPs when extracted from the furnace to the room environment, this remaining Si must be segregated from the NP, as Si greatly reduces its solubility in Au at room temperature **(e)**. Since the abrupt change of temperature occurs in ambient atmosphere rich in oxygen, the remaining Si that is segregated from the NP is immediately exposed to oxygen when its temperature is still relatively high, which produces its rapid oxidation **(f)**. This mechanism is compatible with the extruded tip morphology observed in the images, so that the extrusion would correspond to the segregation process of the Si remaining in the alloy NPs during cooling. The observation of NPs with different degree of extrusion is attributed to a different initial amount of Si remaining at the alloy NPs. This explanation is also consistent with the structures previously observed by TEM at the tips of Si NW that were exposed to a controlled thermal oxidation process by oxygen flow at high temperatures, which resulted in general in thick silica encapsulating layers with different morphologies depending on the oxidation conditions. The results of this work reveal that even without the use of an oxygen flow, the NW tips are susceptible to form Au-SiO_2 core-shell nanostructures.
